# Hypolipidemic effect of XH601 on hamsters of Hyperlipidemia and its potential mechanism

**DOI:** 10.1186/s12944-017-0472-z

**Published:** 2017-05-02

**Authors:** Meng-Jie Zhao, Shan-Shan Wang, Yao Jiang, Ying Wang, Hong Shen, Pei Xu, Hua Xiang, Hong Xiao

**Affiliations:** 1 Nanjing Medical University, Affiliated Nanjing Brain Hospital, No. 264 Guangzhou Road, Nanjing, Jiangsu 210029 People’s Republic of China; 2 Department of Pharmaceutical Chemistry, China Pharmaceutical University, No. 24 Tong Jia Xiang, Nanjing, China

**Keywords:** XH601, Hyperlipidemia, Golden Syrian hamster, 3 T3-L1 adipocyte, PPARs

## Abstract

**Background:**

The novel compound XH601 is a synthesized derivative of formononetin. The present study was to investigate the hypolipidemia effect and potential mechanism of XH601.

**Methods:**

Male Golden Syrian hamsters were induced by high-fat diet (HFD) for eight weeks and the hyperlipidemic model was established successfully. After XH601 treatment, serum and hepatic biochemistry parameters of hamsters were detected and the effect of XH601 on adipose tissue was also analyzed. Furthermore, 3 T3-L1 cell differentiation by Oil-Red-O staining was observed and the mRNA and protein expression of peroxisome proliferator-activated receptors (PPARs) were measured by qRT-PCR and Western-blot in mature adipocytes.

**Results:**

The in vivo results suggest that XH601 significantly decreased the adipose weight and levels of serum triglyceride (TG), total cholesterol (TC), low-density lipoprotein (LDL-C), apolipoprotein B (Apo-B), apolipoprotein E (Apo-E), while increased serum high-density lipoprotein (HDL-C). The in vitro results implied that XH601 up-regulated the mRNA and protein expression of both PPARα and PPARβ/δ in a dose-dependent manner.

**Conclusions:**

The study suggests that XH601 exhibited strong ability to improve the dyslipidemia in hamsters fed with high-fat diet. The potential mechanism of XH601 was associated with the up-regulation of PPARα and PPARβ/δ mRNA and protein expression.

**Electronic supplementary material:**

The online version of this article (doi:10.1186/s12944-017-0472-z) contains supplementary material, which is available to authorized users.

## Background

Cardiovascular diseases are the leading cause of both death and disability around the world [1] and hyperlipidemia is considered to be one of the greatest risk factors contributing to the prevalence and severity of atherosclerosis and subsequent coronary heart disease [2]. Hyperlipidemia is some kind of metabolic syndrome, due to dietary factors and a sedentary lifestyle, and is commonly characterized by elevated serum total cholesterol and triglycerides [3]. For over 20 years, statins have been utilized as one of the most widely prescribed medications for treatment of dyslipidemia [4]. Despite their highly beneficial effects in clinical practice, most commonly seen side effects of statins have been reported, including mild-to-moderate elevations in liver transaminases [5] and other adverse effects including neurological damage, myopathy, and an increased risk of diabetes [6–9]. Recently, alternative therapeutics of herbs and natural agents are applied for patients with metabolic syndrome [10].

Over the past several decades, a pronounced increase of interest in the research of physiologic and pharmacologic effects of naturally bioactive compounds has taken place. Data obtained from preclinical animal experiments as well as clinical and epidemiological studies suggest that isoflavones may prevent dyslipidaemia, obesity, atherosclerosis, type 2 diabetes, nonalcoholic fatty liver disease, etc. [11–13] Formononetin is an O-methylated isoflavone present in different bean types at various levels. Previously, extracts containing formononetin have been investigated and are proved to have positive influences on dyslipidemia [14]. Peroxisome proliferator-activated receptors (PPARs) are ligand-dependent factors that control energy homeostasis by modulating lipid and carbohydrate metabolism [15]. Research have determined the balanced PPARs activity of formononetin, which may enhance the attractiveness of formononetin in the treatment of metabolic syndrome [16].

The golden Syrian hamster has been increasingly utilized to do research on lipoprotein metabolism and to investigate the effects of hypolipidemic agents such as PPAR agonists, statins and CETP inhibitors [17] because of its similarity to human in terms of lipid utilization and high susceptibility to dietary cholesterol [18]. The main plasma cholesterol carrier of hamsters is LDL, and hamster LDL receptor gene has been isolated and characterized [19] by strong similarities to that of humans. Hamsters synthesize bile acids and hepatic cholesterol and also respond to dietary lipids like humans, thus they are prone to hypercholesterolemia induced by excessive dietary cholesterol intake, while rats are resistant to this [20]. Furthermore, hamsters resemble human beings in lipoprotein metabolism as well [21]. Hence, Syrian hamsters have been utilized for the observation of numerous pharmacologic agents with varying mechanisms of action [22].

**Fig. 1:**
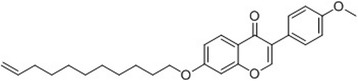
Structural formula of XH601

The XH601 [3-(4-methoxyphenyl)-7-(undec-10-en-1-yloxy)-4H–benzopyran-4-one] (Fig. 1), is a newly synthesized analogue of formononetin. It was provided by the Department of Pharmaceutical Chemistry, China Pharmaceutical University. In the present study, the hypolipidemic effect of XH601 was evaluated in vivo on a high-fat diet-induced hyperlipidemic hamster model [23]. Meanwhile, the potential mechanism was studied in vitro by detecting the relative mRNA and protein expression of PPARs in 3 T3-L1 adipocytes.

## Methods

### Drug preparation

The compound XH601 was provided by China Pharmaceutical University. The initial dose of atorvastatin (Lipitor, Pfizer) for humans is 10 mg per day. The hamster dose was calculated by the human equivalent dose (HED) on the basis of body surface area: assuming a human weight of 60 kg, the HED for 10 (mg)/60 (kg) = 0.17 × 7.4 = 1.23 mg/kg; the coefficient 7.4 was utilized to account for distinctions between hamster and human in body surface area [24, 25].

### Animals and treatment

A total of 50 male Golden Syrian hamsters (90-100 g body weight) were purchased from Beijing Vital River Laboratory Animal Technology Co.,Ltd. (Beijing, China). After acclimation for one week, the hamsters were randomly divided into two groups: normal-fat diet (NFD) group (*n* = 10) and high-fat diet (HFD) group (*n* = 40). The HFD was composed of 10% lard, 2% cholesterol, 0.5% sodium deoxycholate and 87.5% regular diet. The food consumption was restricted to 10 mg/day/hamster. After 8 weeks, the hyperlipidemic hamsters were subdivided into 4 groups (*n* = 10 in each group). (1) HFD group: HFD with vehicle (soybean oil) treatment; (2) ATO group: HFD with 1.23 mg/kg/day atorvastatin; (3) Low-dose XH601 group: HFD with 20 mg/kg/day XH601; (4) High-dose XH601 group: HFD with 50 mg/kg/day XH601. The vehicle group received the same volume of solution equivalent to body weight. Vehicle and drugs were orally administered to hamsters by gastric intubation once a day for 8 weeks.

All animal experiments were approved by the Committee on the Medical Ethics of Nanjing Brain Hospital, affiliated to Nanjing Medical University (Permit Number: 2011KY45), and followed National Research Council Guidelines.

### Sample collection

During the experimental period, body weights were recorded every two weeks. Blood from each hamster was collected from the retro-orbital sinus with a capillary tube after an over-night fasting. Serum was obtained after the blood was centrifuged (8000 rpm at 4 °C for 15 min) and stored at −80 °C until analysis. The animals were sacrificed by cervical dislocation and tissues were harvested, weighed, snap frozen in liquid nitrogen and stored at −80 °C until use.

### Serum and hepatic biochemistry parameters analysis

The serum total cholesterol (TC), triglycerides (TG), high-density lipoprotein cholesterol (HDL-C), low-density lipoprotein cholesterol (LDL-C), apolipoprotein B (Apo-B), apolipoprotein E (Apo-E), aspartate aminotransferase (AST), alanine aminotransferase (ALT) and hepatic TC and TG were measured by enzymatic colorimetric methods using commercial kits (Nanjing Jiancheng Bioengineering Institute, Nanjing, China) following the manufacturer’s instructions.

### Histological analysis

Liver and adipose tissues were isolated from hamsters, fixed in 10% formalin, and embedded in paraffin. Sections were abtained and later stained with hematoxylin and eosin (H&E) for the histological examination. The frozen sections of liver were rinsed with distilled water, stained with 0.2% Oil-Red O and 60% isopropanol for 10 min at 37 °C, and then rinsed again with distilled water. Tissue sections were then observed with a microscope.

### Culture of 3 T3-L1 preadipocytes and stimulation

3 T3-L1 preadipocytes were obtained from Nanjing KeyGEN Biotech Co., Ltd. (Nanjing, China). The cells were cultured in Dulbecco’s modified Eagle’s medium (DMEM) supplemented with 10% fetal bovine serum (FBS) at 37 °C in a humidified incubator under 5% CO2. Adipocytic differentiation was induced by DMEM containing 10% FBS, 10 mg/L insulin, 0.5 μM isobutylmethylxanthine and 1 μM dexamethasone for 48 h. The cells were then maintained in media supplemented with 10% FBS and 10 mg/L insulin for another 48 h, followed by incubating with drugs at different concentrations for 48 h. 3 T3-L1 mature adipocytes monolayers treated by different concentrations of XH601 (1 × 10^−7^, 1 × 10^−6^, 1 × 10^−5^ and 1 × 10^−4^ M) were washed three times with phosphate-buffered saline (PBS) and fixed for 2 h with 3.7% formaldehyde in PBS. Oil-Red O (0.5%) in isopropanol was diluted with 2/3 volumes of water, filtered and added to the fixed cell monolayers for 2 h at room temperature. The cell monolayers were then washed with PBS, and the stained droplets in the cells were visualized. Image of the stained lipid droplets were collected on a microscope. Red staining reveals lipid droplets in the cytoplasm, indicating adipocyte differentiation.

### RNA isolation and quantitative real-time PCR

3 T3-L1 adipocytes were incubated with atorvastatin (10 μM) or XH601 at different concentrations (1 nM, 0.1 μM and 10 μM) for 48 h before isolation of total RNA. Total RNA was isolated using Trizol Reagent. The RNA was reverse transcribed into cDNA using the Takara PrimeScript RT Master Mix (Code No. RR036A). The mRNA expression levels of PPARs were evaluated by qRT-PCR analysis using the FastStart Univeral SYBR Green Master (ROX) (Roche, Germany). qRT-PCR was performed on Eppendorf Mastercycler ep realplex (Eppendorf, Germany). The sequences for primers are listed in Table 1. Target mRNA expression in each sample was normalized to the housekeeping gene GAPDH. The 2^-ΔΔCt^ method was used to calculate relative mRNA expression levels.

**Table 1:** The sequences of the primers used for real-time PCR

Gene name	Forward/reverse primers
PPARα	5′-TGAGGAAGCCGTTCTGTGAC-3’
	5′-GGTGTCATCTGGATGGTTGC-3’
PPARβ/δ	5′-GCCTCGGGCTTCCACTAC-3’
	5′-AGATCCGATCGCACTTCTCA-3’
PPARγ	5′-CCCCTGCTCCAGGAGATCTAC-3’
	5′-GCAATCAATAGAAGGAACACGTTGT-3’

### Western blot analysis

Total proteins were extracted from the adipose tissue and adipocytes, using protein extraction kit (BestBio, Shanghai, China) and RIPA lysis buffer (50 mM Tris-HCl, 150 mM NaCl, 1% NP-40, pH 7.4) containing 5% PMSF, respectively. Protein concentrations were determined by BCA protein assay kit (Nanjing KeyGEN Biotech Co., Ltd., China). Equal amounts of proteins (40 μg) were separated on 10% SDS-polyacrylamide gel and transferred to PVDF membrane. After blocking in 5% skim milk for 1 h, the membranes were probed with primary antibodies (diluted 1:1000) against PPARs and β-Actin (Abcam, Cambridge, MA, USA) overnight at 4 °C. Then the membranes were probed with secondary antibody coupled to horseradish peroxidase. Immunoreactive bands were visualized by using an enhanced chemiluminescence kit (Thermo) and were quantified using the Quantity One software (Bio-Rad Laboratories, UK).

### Statistical analysis

Values are presented as the mean ± S.E.M. Significant differences between diets or treatment groups were determined with SPSS 19.0 software (SPSS Inc., Chicago, IL, USA) using two-tailed Student t-test or one-way ANOVA with Dunnett’s posttest. *P* < 0.05 was considered statistically significant.

## Results

### Effect of XH601 on body mass of hamsters

**Fig. 2:**
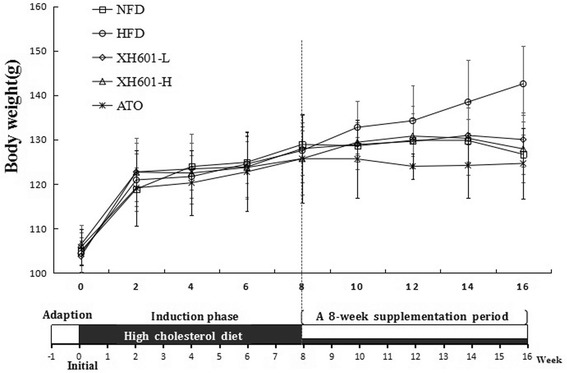
Effect of XH601 on body mass of hamsters. In the induction phase, hamsters were separated into the NFD group (*n* = 10) and HFD group (*n* = 40). After 8 weeks, the 40 hamsters were randomly assigned to four groups (ten hamsters/group) for solvent, XH601 (20 mg/kg/day or 50 mg/kg/day) or ATO (1.23 mg/kg/day) administration

The body mass of hamsters are in Table 2. There were no differences in initial body weight and average daily food intake among all dietary groups in the experiment. After a 16-week induction, the body weights of hamsters in HFD group significantly increased (*P* < 0.05) comparing with those fed a normal diet (Fig. 2). Compared with the vehicle control, a significant decrease in the body weight was detected in the ATO group (*P* < 0.05) since drug administration, and XH601 at doses of 20 mg/kg and 50 mg/kg resulted in a significant reduction in body weight gain of 31.18% (*P* < 0.01) and 43.47% (*P* < 0.01), respectively.

**Table 2:** Effect of XH601 on body mass of hamsters (*n* = 10)

Time point	NFD	HFD	XH601-L	XH601-H	ATO
Week-0	104.95 ± 3.13	104.40 ± 4.69	103.76 ± 3.59	106.34 ± 4.39	105.81 ± 4.09
2	118.88 ± 4.89	120.94 ± 5.86	122.74 ± 6.56	122.68 ± 7.71	119.14 ± 8.53
4	123.95 ± 5.30	121.69 ± 4.81	123.42 ± 7.83	122.49 ± 6.83	120.28 ± 7.33
6	124.90 ± 4.69	124.53 ± 7.40	123.87 ± 7.09	123.74 ± 7.02	122.76 ± 8.86
8	128.92 ± 3.16	127.54 ± 5.42	127.97 ± 7.51	125.72 ± 8.15	125.71 ± 9.94
10	128.61 ± 2.04	132.77 ± 5.90	128.92 ± 5.49	129.42 ± 4.41	125.64 ± 8.75
12	129.84 ± 6.13	134.25 ± 7.98	129.61 ± 5.24	130.80 ± 6.72	124.00 ± 2.94
14	129.81 ± 4.89	138.47 ± 9.50	130.95 ± 6.25	130.30 ± 8.25	124.20 ± 7.23
16	126.70 ± 4.48	142.56 ± 8.43^#^	130.02 ± 6.15^*^	127.92 ± 7.61^*^	124.65 ± 7.95^*^
Weight gain	21.75 ± 6.09	38.16 ± 9.82^#^	26.26 ± 4.72^*^	21.57 ± 7.08^**^	18.84 ± 4.86^**^

^#^ *P* < 0.05 versus NFD; ^*^ *P* < 0.05, ^**^ *P* < 0.01 versus HFD by one-way ANOVA with Dunnett’s posttest

### Effect of XH601 on serum lipid profiles

As presented in the following figures, administration of hypercholesterolemic diet to hamsters for 8 weeks resulted in a profoundly elevation in serum TC, TG, LDL-C, HDL-C, Apo-B and Apo-E levels compared with the sham group, indicating that a hyperlipidemic hamster model had been induced successfully [26].

**Fig. 3:**
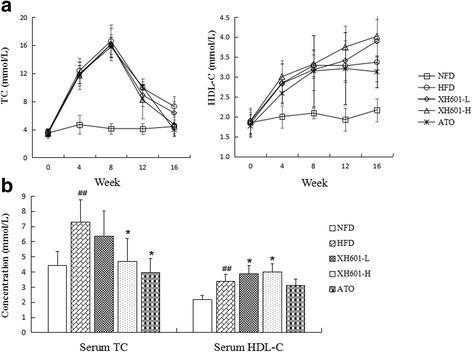
Effect of eight-week XH601 treatment on lipid profiles. **a** Variations on serum TC and HDL-C levels in hyperlipidemic hamsters throughout the experiment. **b** Effect of eight-week drug treatment on serum TC and HDL-C levels. ^##^ *P* < 0.01 versus NFD; ^*^ *P* < 0.05 versus HFD group by one-way ANOVA with Dunnett’s posttest. TC: total cholesterol; HDL-C: high-density lipoprotein cholesterol

At eight weeks after administration of XH601 or ATO, serum TC and HDL-C levels significantly differed among groups, as shown in Fig. 3. TC concentrations were lower with ATO and XH601-H than with HFD alone by 45.54% (*P* < 0.05) and 35.39% (*P* < 0.05), respectively. And at the end of the experiment, serum HDL-C levels of XH601-L and XH601-H groups were higher than HFD group by 1.16-fold (*P* < 0.05) and 1.19-fold (*P* < 0.05), respectively.

**Fig. 4:**
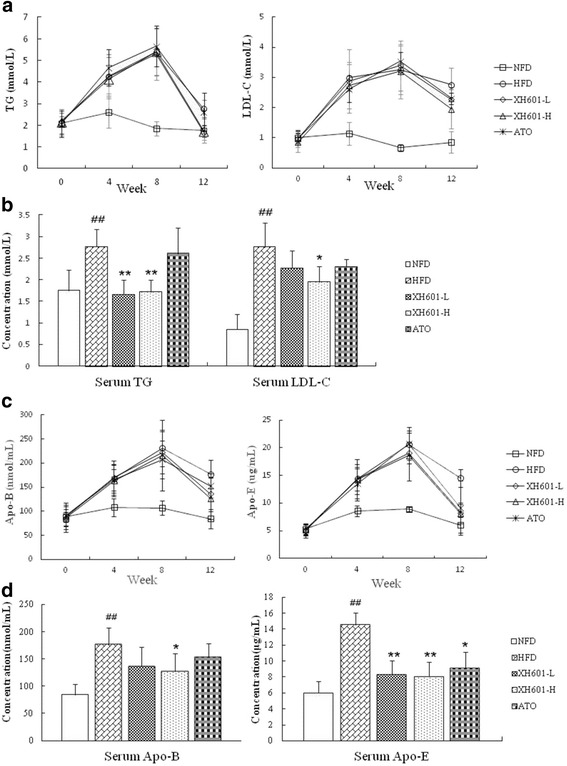
Effect of four-week XH601 treatment on serum profiles. **a** Variations on serum TG and LDL-C levels in hyperlipidemic hamsters throughout the experiment. **b** Effect of four-week drug treatment on serum TG and LDL-C levels. **c** Variations on serum Apo-B and Apo-E levels in hyperlipidemic hamsters throughout the experiment. **d** Effect of four-week drug treatment on serum Apo-B and Apo-E levels. ^##^ *P* < 0.01 versus NFD; ^*^ *P* < 0.05, ^**^ *P* < 0.01 versus HFD group by one-way ANOVA with Dunnett’s posttest. TG: triglyceride; LDL-C: low-density lipoprotein cholesterol; Apo-B: apolipoprotein B; Apo-E: apolipoprotein E

As presented in Fig. 4, hamsters’ serum TG concentration showed significant difference among experimental groups after 4-week administration of ATO or XH601. TG levels were lower with XH601-L and XH601-H than HFD group by 40.07% (*P* = 0.001) and 37.91% (*P* = 0.002). Compared with animals fed a high-cholesterol diet, XH601 at dose of 50 mg/kg significantly decreased serum LDL-C and Apo-B levels by 29.35% (*P* < 0.05) and 28.25% (*P* < 0.05), respectively. At four weeks after drug administration, hamsters serum Apo-E levels of ATO, XH601-L and XH601-H groups declined than HFD group by 37.22% (*P* < 0.05), 42.80% (*P* < 0.05) and 44.18% (*P* < 0.01), respectively.

### Effect of XH601 on hepatic lipid levels

**Fig. 5:**
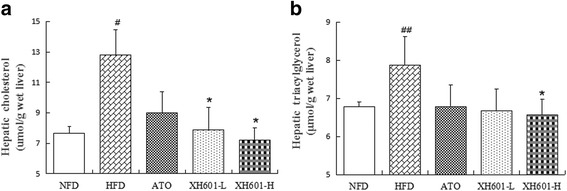
Effect of XH601 on hepatic lipid levels. **a** Effect of eight-week drug treatment on hepatic TC levels. **b** Effect of eight-week drug treatment on hepatic TG levels. ^#^ *P* < 0.05, ^##^ *P* < 0.01 versus NFD; ^*^ *P* < 0.05 versus HFD group by one-way ANOVA with Dunnett’s posttest. TC: total cholesterol; TG: triglyceride

Liver TC content significantly differed among groups and was higher with HFD alone, by 1.67-fold (*P* < 0.05), than controls (Fig. 5a). Furthermore, hepatic TC level was lower with XH601-L and XH601-H than HFD by 38.33% (*P* < 0.05) and 43.71% (*P* < 0.05), respectively. Hepatic TG level of HFD group was pronounced increased comparing with NFC group, and XH601 at 50 mg/kg/day markedly declined the concentration of TG (*P* < 0.05).

### Effect of XH601 on hepatic dysfunction

**Fig. 6:**
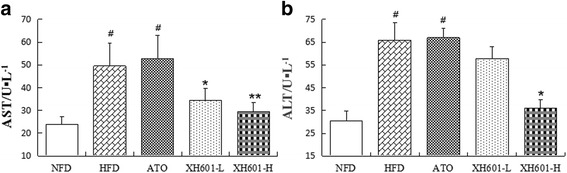
Effect of XH601 on hepatic dysfunction. **a** Effect of eight-week drug treatment on serum AST levels. **b** Effect of eight-week drug treatment on serum ALT levels. ^#^ *P* < 0.05 versus NFD; ^*^ *P* < 0.05, ^**^ *P* < 0.01 versus HFD group by one-way ANOVA with Dunnett’s posttest. AST: aspartate aminotransferase; ALT: alanine aminotransferase

Concerning the side effects of statins on significantly elevating liver enzyme levels, we examined the serum AST and ALT concentrations of hamsters. As presented in Fig. 6, both AST and ALT levels of HFD group and ATO group obviously increased comparing to NFD group, while eight-week of low-dose XH601 administration (20 mg/kg) resulted in a profoundly decrease in AST than HFD group by 30.24% (*P* < 0.05). Meanwhile, eight-week of XH601 treatment at dose of 50 mg/kg significantly declined AST and ALT levels by 40.28% (*P* < 0.01) and 45.20% (*P* < 0.05), respectively.

### Effect of XH601 on adipose and liver tissue

Hamsters were sacrificed after 8 weeks of XH601 administration; liver, kidney, total fat and epididymal fat pad (EFP) were removed and tissue weights were measured and calculated the percentages of the whole body weight individually. As shown in Table 3, the relative EFP, total fat and liver weight were marked higher in HFD group than control. The relative EFP weight was lower with XH601-L and XH601-H than HFD alone by 15.04% (*P* < 0.05) and 13.82% (*P* < 0.05), respectively. Compared with the HFD group, the relative total fat weight of hamsters in XH601-L and XH601-H group significantly declined by 17.30% (*P* < 0.05) and 16.82% (*P* < 0.05). Meanwhile, the relative liver weight of hamsters in XH601-H group profoundly decreased by 16.87% (*P* < 0.05).

**Table 3:** Tissue weights of hamsters at the end of the experiment (*n* = 10)

Tissue Weights	NFD	HFD	ATO	XH601-L	XH601-H
EFP (g)	2.10 ± 0.54	3.47 ± 0.42^##^	2.34 ± 0.61^**^	2.57 ± 0.35^**^	2.54 ± 0.41^**^
Relative EFP (%)	1.68 ± 0.34	2.46 ± 0.17^##^	2.00 ± 0.22^*^	2.09 ± 0.23^*^	2.12 ± 0.25^*^
Total fat (g)	3.19 ± 0.85	5.70 ± 1.33^##^	4.68 ± 1.13	4.47 ± 0.59^*^	4.32 ± 0.62^*^
Relative fat (%)	2.55 ± 0.57	4.22 ± 0.55^##^	4.00 ± 0.36	3.49 ± 0.52^*^	3.51 ± 0.59^*^
Liver (g)	4.46 ± 0.45	5.61 ± 0.70^#^	5.59 ± 0.54	4.77 ± 0.68	4.63 ± 0.93^*^
Relative liver (%)	3.60 ± 0.41	4.03 ± 0.46^#^	4.87 ± 0.51	3.68 ± 0.37	3.35 ± 0.28^*^
Kidney (g)	0.86 ± 0.04	0.85 ± 0.09	0.96 ± 0.05	0.84 ± 0.06	0.90 ± 0.13
Relative kidney (%)	0.70 ± 0.03	0.61 ± 0.02	0.84 ± 0.17^**^	0.64 ± 0.03	0.68 ± 0.06

^#^ *P* < 0.05, ^##^ *P* < 0.01 versus NFD; ^*^ *P* < 0.05, ^**^ *P* < 0.01 versus HFD by one-way ANOVA with Dunnett’s posttest. EFP: Epididymal fat pad

### Histological assessment

**Fig. 7:**
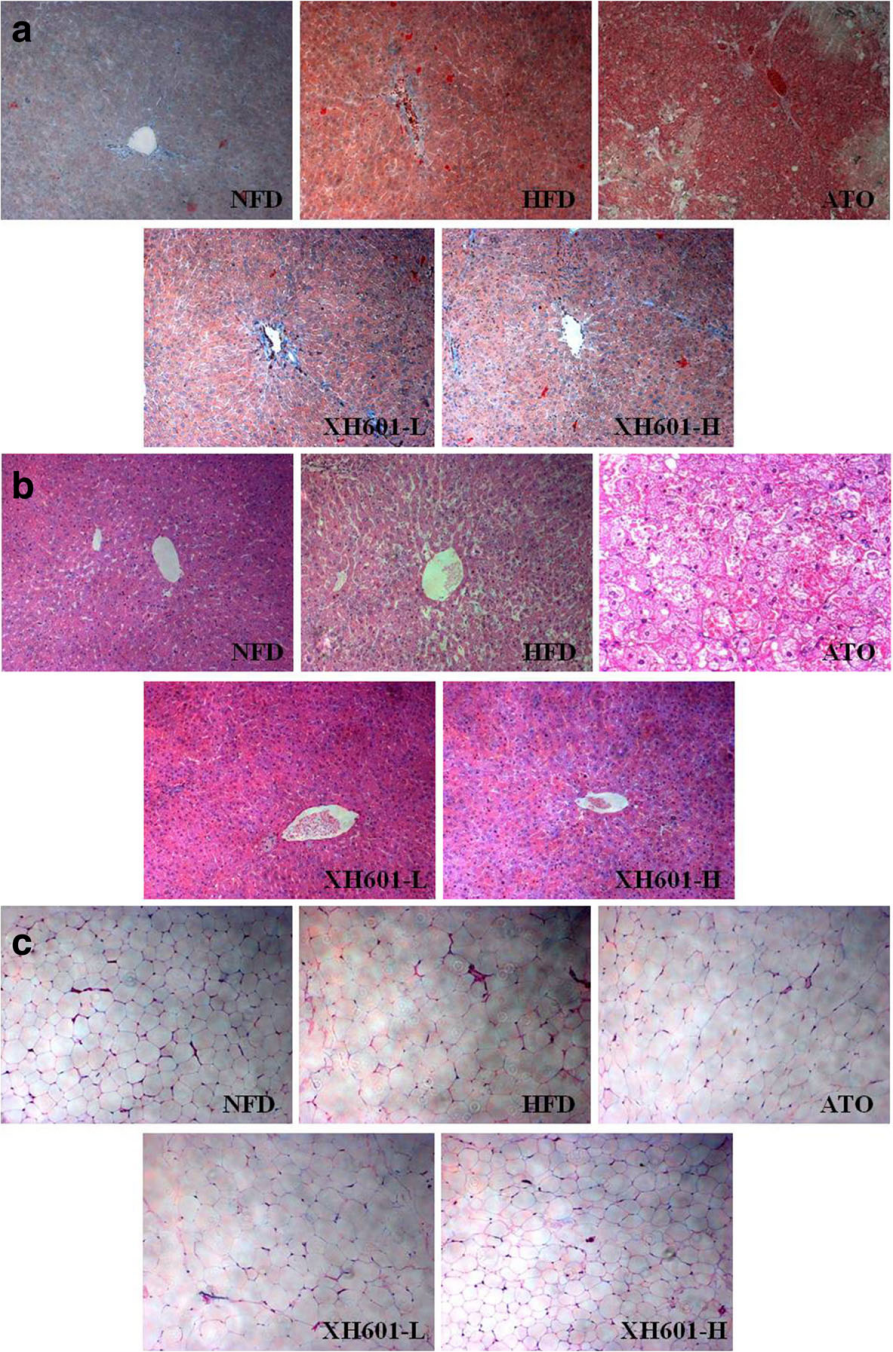
Effect of XH601 on histology of liver and adipose tissue of hamsters (200╳). **a** and **b** Photomicrograh of liver examination based on Oil-Red O staining and H&E staining, respectively. **c** Photomicrograh of adipose tissue examination based on H&E staining

In the liver section of the normal group, hepatic lobular architecture remained clear and intact without any abnormalities. Lipid deposits as macrovesicular and microvesicular steatosis were abundant in the section of the HFD group hamsters. It was shown that treatment with XH601 dose-dependently repressed these changes induced by high-fat diet and ameliorated the symptoms of fatty liver (Fig. 7b), with less lipid droplets in the Oil-Red O staining sections (Fig. 7a). While the histological pictures showed that liver tissue was damaged in ATO administrated hamsters. Meanwhile, histological analysis also showed that adipocyte size in adipose tissue was smaller in the XH601 groups than in the control group fed a high-fat diet (Fig. 7c).

### Effect of XH601 on adipocyte differentiation

**Fig. 8:**
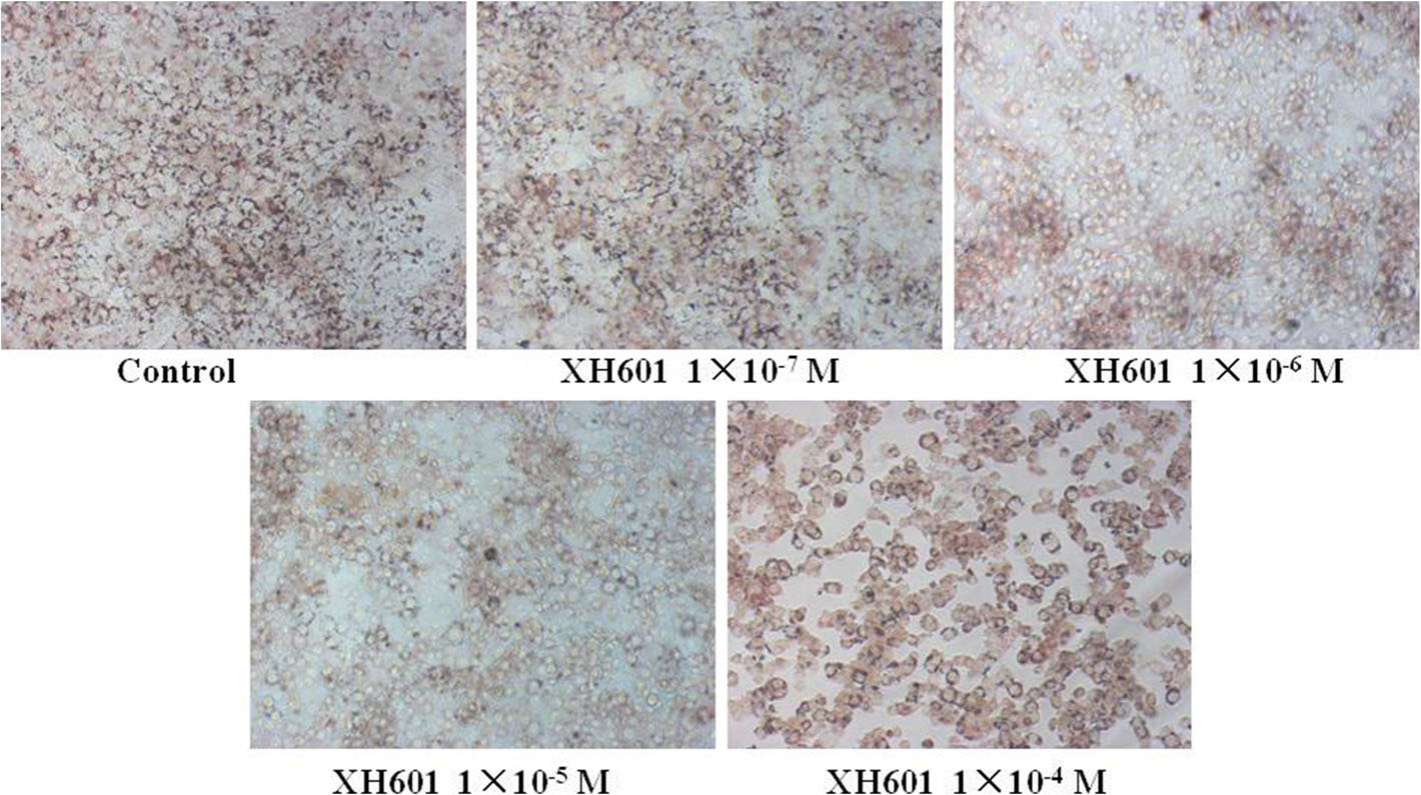
Effect of XH601 on adipocyte differentiation. 3 T3-L1 mature adipocytes monolayers were treated by different concentrations of XH601 (1 × 10^−7^, 1 × 10^−6^, 1 × 10^−5^ and 1 × 10^−4^ M) for 48 h and were fixed and stained with the lipophilic dye Oil-Red-O. Lipid droplets were visualized by Oil-Red-O staining

Differentiation of 3 T3-L1 preadipocytes can be induced into adipocytes in cell culture. They varied from an extended fibroblast-like morphology into a round one with cytoplasmic lipid vesicles containing newly biosynthesized triglyceride after differentiation. In this study, the effect of XH601 on adipogenic differentiation was investigated at four concentrations (1 × 10^−7^, 1 × 10^−6^, 1 × 10^−5^ and 1 × 10^−4^ M) in confluent 3 T3-L1 cells. Differentiation of 3 T3-L1 cell was characterized using Oil-Red-O staining for quantification through spectrophotometric analysis with isopropanol. As is shown in Fig. 8, lipid accumulation in 3 T3-L1 mature adipocytes significantly and dose-dependently decreased after drug treatment and XH601 showed the inhibitory effect on this adipogenic differentiation.

### Effect of XH601 on PPARs mRNA and protein expression

**Fig. 9:**
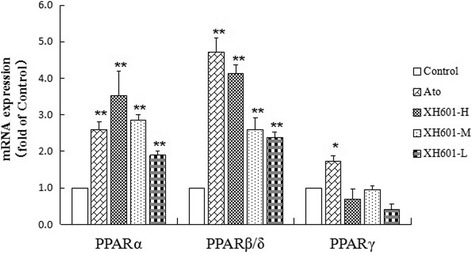
Effect of XH601 on PPARs mRNA expression in 3 T3-L1 adipocytes. ^*^ *P* < 0.05, ^**^ *P* < 0.01 versus control by one-way ANOVA with Dunnett’s posttest. ATO: atorvastatin treatment at 10 μM in cells; XH601-H: XH601 treatment at 10 μM in cells; XH601-M: XH601 treatment at 0.1 μM in cells; XH601-L: XH601 treatment at 1 nM in cells

In this study, the hypolipidemic effects of XH601 in vivo has been determined, to further charify the mechanism of XH601, the effects of XH601 on PPARs expression were measured on 3 T3-L1 adipocytes in vitro. As presented in Fig. 9, atorvastatin significantly increased PPARα, PPARβ/δ and PPARγ mRNA expression by 2.6-, 4.71- and 1.72-fold than control (*P* < 0.01), respectively. Compared to the control, XH601 profoundly up-regulated the mRNA expression of both PPARα and PPARβ/δ (*P* < 0.01) in a dose-dependent manner, while showed no obvious effect on PPARγ.

**Fig. 10:**
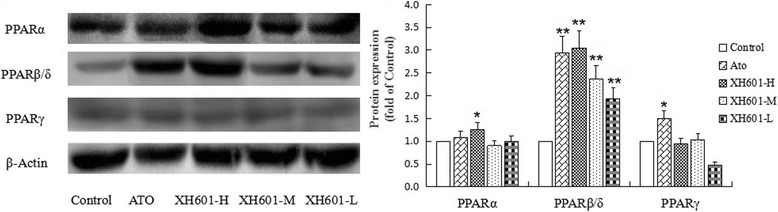
Effect of XH601 on PPARs protein expression in 3 T3-L1 adipocytes. ^*^ *P* < 0.05, ^**^ *P* < 0.01 versus control by one-way ANOVA with Dunnett’s posttest. ATO: atorvastatin treatment at 10 μM in cells; XH601-H: XH601 treatment at 10 μM in cells; XH601-M: XH601 treatment at 0.1 μM in cells; XH601-L: XH601 treatment at 1 nM in cells

**Fig. 11:**
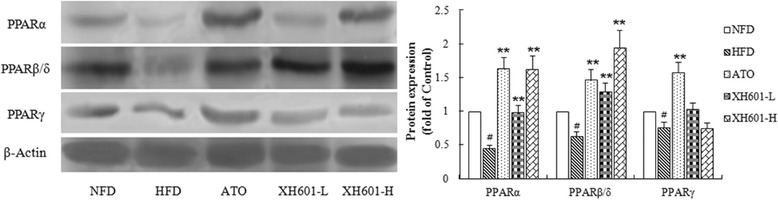
Effect of XH601 on PPARs protein expression in adipose tissue. ^#^ *P* < 0.05 versus NFD; ^*^ *P* < 0.05, ^**^ *P* < 0.01 versus HFD by one-way ANOVA with Dunnett’s posttest

As shown in Fig. 10 and Fig. 11, the protein expression of PPARβ/δ and PPARγ after atorvastatin treatment was significantly increased. By contrast, the protein expression of PPARα was profoundly up-regulated by high-dose XH601 treatment (*P* < 0.05), while XH601 markedly improved PPARβ/δ protein expression (*P* < 0.01). The effects of XH601 on the protein expression of PPARs were roughly in accordance with its effect on the mRNA expression of them.

## Discussion

The clinically significant consequences of hyperlipidemia include two life-threatening conditions, pancreatitis and atherosclerosis. Atherosclerosis is responsible for a large proportion of cardiovascular diseases, which are the leading cause of death in industrialized countries [26]. Additionally, many metabolic diseases, such as obesity and diabetes, are comorbidities associated with dyslipidemia. Drugs that attenuate dyslipidemia are acutely important in the prevention of cardiovascular diseases [27]. By activating PPARs system, statins have been advocated to play a major role in stabilization and regression of lipid-rich plaques [28–30]. However, the adverse effects of these drugs limit their more widely using and, thus, discovery and characterization of novel scaffolds based small molecules are expected to provide a superior profile in dyslipidemia intervention [20].

In the present study, we demonstrated the hypolipidemic properties and mechanism of XH601 modified from formononetin for the first time. The significant lipid-lowering effects of XH601 were observed in serum and hepatic lipid parameters. Moreover, our data indicated that XH601 decreased the weight of total adipose tissue, while atorvastatin not. To correspond to the hypolipidemic effects of XH601 on hamsters, its potential mechanism was also investigated in vitro [31]. Adipocytes play a significant role in lipid homeostasis and energy balance by triglyceride storage and free fatty acids release. In addition, adipocytes secrete dozens of factors which participate in energy metabolism of adipose tissues [32]. Thus, we used 3 T3-L1 adipocytes to explore the effects of XH601 on adipogenesis. Oil red O stain of mature differentiated 3 T3-L1 cells indicated that XH601 reduced the accumulation of lipid droplets and decreased lipid content. These results showed that XH601 may play a positive role in the prevention of adipogenesis.

Considerable research effort has focused on isoflavones as the main hypolipidemic agent in soy because of their antioxidative and mild estrogenic activity. One relevant mechanism may be by PPARs, nuclear receptors that participate in cellular lipid homeostasis and insulin action [33]. PPARs are also transcriptional activators of genes encoding enzymes to metabolize fatty acid in the liver as well as other metabolic tissues [34] and atorvastatin has been demonstrated to exert cardiac protective influences by pronouncedly up-regulating PPARs expression [35–37]. In our research, relative protein expression of PPARs and phosphorylated PPARs (Additional file 1) were observed in 3 T3-L1 adipocytes and adipose tissues of hamsters, thereby investigating the potential mechanism of XH601.

PPARα is a transcription factor that regulates the metabolism of lipids, carbohydrates, and amino acids and is activated by ligands such as drugs used to treat dyslipidemia [38]. By elevating lipoprotein lipase (LPL), triglyceride-VLDL lipolysis was increased and VLDL clearance was improved. Thereby atherogenic LDL-C, serum and hepatic TG were reduced. In addition, energy uncoupling was simultaneously induced in adipose tissue [20]. We found that XH601 activated PPARα more efficiently than atorvastain in adipocytes. Correspondingly, in the high-fat diet-induced hyperlipidemic hamsters, 4-week treatment with XH601 significantly lowered serum LDL-C, TG and hepatic TG levels more potently than atorvastatin. Therefore, XH601 showed stronger hypolipidemic effect when comparing with atorvastatin.

Moreover, there have been several tantalizing clues that PPARβ/δ may also modulate aspects of lipid homeostasis [39] and it can be proposed as a possible pharmacological target for the treatment of obesity, IR, and dyslipidemia in order to contribute the potent vascular antiatherogenic effects [40]. PPARβ/δ activation inhibits human macrophage foam cell formation and inflammation induced by VLDL and it also exerts favorable effects on HDL-C level [41]. Moreover, relevant study showed that PPARβ/δ agonist treatment decreased plasma TG, Apo-B, total and LDL-cholesterol [42]. Our data suggested that XH601 up-regulated PPARβ/δ almost at the same level as atorvastatin. And XH601 treatment significantly decreased serum TC, Apo-B and increased HDL-C level in hamsters. So the elevated expression of PPARβ/δ had a favorable role in XH601 treatment to combat hyperlipidemia.

The molecular mechanism of the role of PPARγ in adipocytes has been explored as well. PPARγ can inhibit the lipolysis by stabilizing the deposited fat with perilipin as well as enhancing the insulin sensitivity [43] by transcriptionally activating genes involved in insulin signaling, glucose uptake and fatty acid uptake and storage [44]. Nevertheless, heart failure, massive weight gain, and appearance of oedema are relevant to PPARγ [45–47]. It has been shown that moderate reduction of PPARγ activity in mice prevented the insulin resistance and obesity induced by a high-fat diet [48]. In the present experiment, swollen spleen was observed after histological dissection. In addition, kidney percentage with ATO was marked higher than HFD, indicating the kidney-related damage of atorvastatin. By contrast, the hyperlipidemic-associated damages were significantly and dose-dependently optimized in the XH601 groups. The results indicated that appropriate functional antagonism of PPARγ may be a logical approach for protection against obesity and obesity-associated diseases. However, further research should be carried out to testify the favorable effects of XH601 on dyslipidemia.

## Conclusions

The obtained data of the in vivo and in vitro studies suggest that XH601 exhibited strong ability to improve the dyslipidemia in hamsters fed with high-fat diet and the hypolipidemic mechanisms of XH601 were associated with the up-regulation of PPARα and PPARβ/δ expression. The findings in our study supports our hypothesis that XH601 could be developed into a pharmacologic agent for hyperlipidemia with potential hepatic-protective effects on liver damages.

## Additional file

Additional file 1: Figure S12. Effect of XH601 on phosphorylated PPARα and PPARγ protein expression in 3 T3-L1 adipocytes. ^*^ *P* < 0.05, ^**^ *P* < 0.01 versus control by one-way ANOVA with Dunnett’s posttest. ATO: atorvastatin treatment at 10 μM in cells; XH601-H: XH601 treatment at 10 μM in cells; XH601-M: XH601 treatment at 0.1 μM in cells; XH601-L: XH601 treatment at 1 nM in cells. **Figure S11.** Effect of XH601 on phosphorylated PPARα and PPARγ protein expression in adipose tissue. ^#^ *P* < 0.05 versus NFD; ^*^ *P* < 0.05, ^**^ *P* < 0.01 versus HFD by one-way ANOVA with Dunnett’s posttest. (DOC 217 kb)

## Abbreviations

ALT: Alanine aminotransferase
Apo-B: Apolipoprotein B
Apo-E: Apolipoprotein E
AST: Aspartate aminotransferase
ATO: Atorvastatin
CETP: Cholesteryl ester transfer protein
DMEM: Dulbecco’s modified Eagle’s medium
EFP: Epididymal fat pad
HDL-C: High-density lipoprotein cholesterol
HED: Human equivalent dose
HFD: High-fat diet
LDL-C: Low-density lipoprotein cholesterol
NFD: Normal-fat diet
PBS: Phosphate-buffered saline
qRT-PCR: Quantitative real time polymerase chain reaction
TC: Total cholesterol
TG: Triglyceride
XH601: 3-(4-methoxyphenyl)-7-(undec-10-en-1-yloxy)-4H–benzopyran-4-one
