# Supplementary material for: Hypolipidemic effect of XH601 on hamsters of Hyperlipidemia and its potential mechanism
Source: Lipids Health Dis. 2017 May 2;16:85. doi: 10.1186/s12944-017-0472-z (PMC5414347; doi:10.1186/s12944-017-0472-z)
Supplement: Additional file 1: Figure S12. — Effect of XH601 on phosphorylated PPARα and PPARγ protein expression in 3 T3-L1 adipocytes. * P < 0.05, ** P < 0.01 versus control by one-way ANOVA with Dunnett’s posttest. ATO: atorvastatin treatment at 10 μM in cells; XH601-H: XH601 treatment at 10 μM in cells; XH601-M: XH601 treatment at 0.1 μM in cells; XH601-L: XH601 treatment at 1 nM in cells. Figure S11. Effect of XH601 on phosphorylated PPARα and PPARγ protein expression in adipose tissue. # P < 0.05 versus NFD; * P < 0.05, ** P < 0.01 versus HFD by one-way ANOVA with Dunnett’s posttest. (DOC 217 kb) [file 12944_2017_472_MOESM1_ESM.doc]

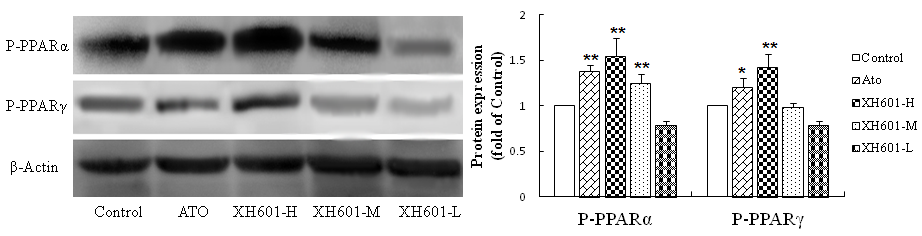


**Figure 12.** Effect of XH601 on phosphorylated PPARα and PPARγ protein expression in 3T3-L1 adipocytes. ^*^P<0.05, ^**^P<0.01 versus control by one-way ANOVA with Dunnett’s posttest. ATO: atorvastatin treatment at 10 μM in cells; XH601-H: XH601 treatment at 10 μM in cells; XH601-M: XH601 treatment at 0.1 μM in cells; XH601-L: XH601 treatment at 1 nM in cells.


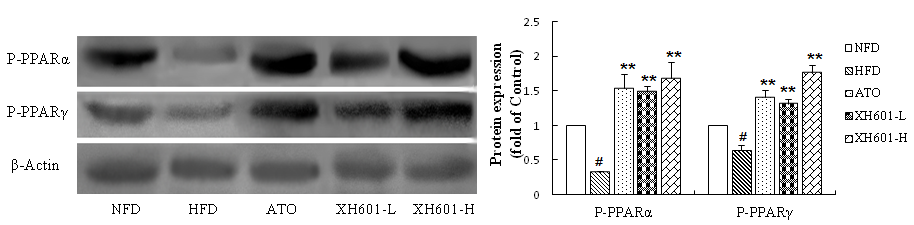


**Figure 11.** Effect of XH601 on phosphorylated PPARα and PPARγ protein expression in adipose tissue. ^#^P<0.05 versus NFD; ^*^P<0.05, ^**^P<0.01 versus HFD by one-way ANOVA with Dunnett’s posttest.
